# Reasons for raising the maximum acceptable daily intake of EDTA and the benefits for iron fortification of foods for children 6–24 months of age

**DOI:** 10.1111/mcn.12110

**Published:** 2014-02-13

**Authors:** Carel Theo Jozef Wreesmann

**Affiliations:** ^1^ Akzo Nobel Functional Chemicals B.V. Amersfoort The Netherlands

**Keywords:** iron, infant iron status, iron deficiency anaemia, EDTA, acceptable daily intake, zinc

## Abstract

The current maximum acceptable daily intake (ADI) of ethylenediaminetetraacetic acid (EDTA) of 1.9 mg day^−1^ per kilogram bodyweight (mg day^−1^ kgbw^−1^) limits the daily intake of iron as iron EDTA [ferric sodium EDTA; sodium iron(III) EDTA] to approximately 2–2.5 mg day^−1^ for children 6–24 months of age. This limit was defined by the Joint FAO/WHO Expert Committee on Food Additives (JECFA) in 1973 based on data from an animal‐feed study published in 1963. Other animal studies indicate that this limit can be raised to 4.4 or possibly up to 21.7 mg day^−1^ kgbw^−1^, which is 2.3–11.4 times higher than the current value. For nearly 50 years, iron EDTA has been used in France in medicinal syrup for infants 1–6 months of age. The maximum recommended dosage of this drug is 37 times higher than the maximum ADI of EDTA. No adverse health effects have been reported as a result of this medicinal consumption of iron EDTA. Raising the maximum ADI of EDTA to only 4.4 mg day^−1^ kgbw^−1^ would enable iron EDTA, an iron fortificant with proven bioavailability in phytate‐rich meals, to be added in adequate amounts to cereal‐based meals for children 6–24 months of age, who are at risk of iron deficiency.

## Introduction

Millions of young children predominantly in developing and emerging countries may not reach their full physical and intellectual potential because of micronutrient deficiencies in their diet (Horton 2008). Among the most prominent micronutrient deficiencies are iron and zinc, and iron especially may adversely affect their long‐term cognitive and emotional development (EFSA 2013; Lozoff *et al*. 2013). Yet, these problems could be overcome with relatively low investment in providing micronutrients through food fortification (Hoddinott *et al*. 2012).

Minerals are often not well absorbed from food because of the presence of phytate, also referred to as phytic acid. It is the plant form of phosphorus and thus an essential component of plant foods. Plant‐based diets are rich in phytate (Gibson *et al*. 2010). This otherwise health‐beneficial food ingredient (Kumar *et al*. 2010) converts mineral ions, such as iron and zinc, to insoluble complexes that obstruct absorption in the gastrointestinal tract (Hurrell 2002). Many food products contain levels of phytic acid in excess of 100 mg per 100 g (Ma *et al*. 2005).

To enable sufficient iron absorption in the absence of enhancing compounds such as ascorbic acid, the phytic acid to iron molar ratio needs to be decreased to less than 1:1 and preferably to lower than 0.4:1 (Hurrell 2004). The molar mass of iron is 56 mg mmol^−1^ and of phytic acid 660 mg mmol^−1^. At a phytic acid level of 100 mg per 100 g (1000 ppm; 1.5 mmol kg^−1^), the level of intrinsic and added iron (except iron EDTA) should be above 85 ppm (1.5 mmol kg^−1^, 8.5 mg per 100 g of food) and preferably above 210 ppm (3.75 mmol kg^−1^, 21 mg per 100 g of food). However, the latter level of added iron could result in organoleptic and possibly also stability issues.

**Figure 1:**
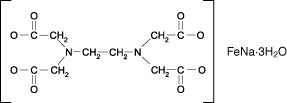
Structural formula of EDTA‐FeNa·3H_2_O (iron EDTA).

Various studies have shown that the inhibitory effect of phytate on iron absorption in cereal foods can be counteracted safely and effectively by iron EDTA (Hurrell *et al*. 2010). This compound is also known as sodium iron(III) EDTA (NaFeEDTA) or ferric sodium EDTA (FeNaEDTA), and in pharmaceutical preparations, it is invariably referred to as sodium feredetate. EDTA stands for ethylenediaminetetraacetic acid (also denoted as H_4_EDTA or EDTA‐H_4_). Ferric sodium EDTA is a yellow to yellow‐brown powder that is highly stable when stored under ambient conditions. In storage‐stable form, iron EDTA carries three molecules of crystal water: EDTA‐FeNa·3H_2_O. In this chemical form, it is obtained via crystallisation and should preferably be used in food applications (FCC 2012). For the structural formula of iron EDTA (EDTA‐FeNa·3H_2_O), see Fig. 1. The function of EDTA in iron EDTA is to ensure adequate uptake of iron by intestinal cells from food rich in phytate. Thus, the EDTA molecules in iron EDTA can be considered to be a vehicle to transport iron. EDTA could also fulfil a similar role with respect to zinc ions (Hess & Brown 2009).

The Joint FAO/WHO Expert Committee on Food Additives (JECFA) classifies iron EDTA as ‘suitable for use as a source of iron for food fortification’ (JECFA 2007). The World Health Organization (WHO) recommends iron EDTA as the only iron compound suitable for fortification of high‐extraction wheat and maize flour, and as one of only four iron compounds for fortification of low‐extraction flours (WHO 2009). Recently, the use of iron EDTA in foods and food supplements was approved in the European Union (EU) (EFSA 2010; EU 2010; EU 2011) after earlier food approvals in China (PRC 1994; PRC 1996), the Philippines (Republic of the Philippines 1995), most Latin American countries by about 2000, United States (FDA 2004; FDA 2006), Australia and New Zealand (FSANZ 2008), and India (Gazette of India 2011). In spite of wide approval, iron EDTA cannot be used in the required amounts in complementary foods for older infants and young children (6–24 months of age) because of concern about exceeding the maximum acceptable daily intake (ADI) of EDTA (Yang *et al*. 2011).

ADI has been defined by JECFA as an ‘estimate of the amount of a food additive [expressed in mg day^−1^ per kilogram bodyweight (mg day^−1^ kgbw^−1^)] that can be ingested daily over a lifetime without appreciable health risk’. The maximum ADI is derived from the highest dose level expressed in mg day^−1^ kgbw^−1^ that can be administered in long‐term animal studies without inducing adverse health effects. This level is divided by a safety margin of usually 100 in order to arrive at a maximum intake level, also expressed in mg day^−1^ kgbw^−1^, that can be deemed safe for humans (WHO 1987).

Ferric sodium EDTA consists of iron, sodium and EDTA, and for each of these three constituents, maximum intake levels are applicable (Munro 1993). In 1983, JECFA proposed a provisional maximum tolerable daily intake for iron in food of 0.8 mg day^−1^ kgbw^−1^ (JECFA 1983). In 2001, the US Institute of Medicine (IOM) published a tolerable upper limit for adults of 45 mg day^−1^ of iron (IOM 2001). The latest WHO recommendation on sodium is a maximum intake of 2 g day^−1^ (WHO 2012a). An intake of 45 mg of iron as iron EDTA is equivalent to an intake of 18 mg of sodium, which is less than 1% of the recommended maximum of 2 g day^−1^.

In 1973, JECFA defined a maximum ADI for calcium disodium EDTA (CaNa_2_EDTA) of 2.5 mg day^−1^ kgbw^−1^ (JECFA 1974). This quantity of calcium EDTA (CaNa_2_EDTA) contains the same number of EDTA molecules (6.7 *μ*mol) as present in 0.375 mg iron as iron EDTA (FeNaEDTA·3H_2_O) or 1.95 mg of EDTA (H_4_EDTA). In a safety re‐evaluation, JECFA redefined the maximum ADI of EDTA to 1.9 mg EDTA (JECFA 2007). This amount is equivalent to 6.5 *μ*mol of EDTA molecules and limits the intake of iron as iron EDTA to 0.37 mg day^−1^ kgbw^−1^.

The current maximum ADI of EDTA (JECFA 1974; JECFA 2007) allows a maximum daily dosage of 2–2.5 mg day^−1^ of iron as iron EDTA for infants and young children (Yang *et al*. 2011), whereas experts advocate that this amount should be two to four times higher (Rosenberg *et al*. 2011). An amount four times higher would meet the recommended iron intake for under‐fives (De Pee *et al*. 2011), but because of high bioavailability, a daily intake of 5 mg iron as iron EDTA may provide enough additional iron.

Vitamin C has also been found to enhance iron absorption from single meals, including those rich in phytate (Diaz *et al*. 2003; Thankachan *et al*. 2008; Cercamondi *et al*. 2013). However, long‐term studies in which vitamin C was added to diets containing foods naturally rich in iron but no fortificant iron did not show a meaningful improvement of the iron status (Cook *et al*. 1984; Hunt *et al*. 1994; Garcia *et al*. 2003). No studies have been found in which vitamin C has been used to improve iron status based on long‐term intake of iron‐fortified food. Furthermore, the instability of vitamin C especially at higher temperatures is considered a drawback to its use in staple food fortification (Teucher *et al*. 2004).

This paper reviews the basis for setting the maximum ADI of EDTA and assesses the safety of a higher level that is deemed adequate for fortifying complementary foods with iron EDTA for children 6–24 months of age. An increase in the maximum ADI of EDTA would widen the scope for iron EDTA in food fortification and also the use of suitable EDTA compounds in food fortification to improve zinc absorption.

### Key messages

- Test animal data support an increase of the current maximum acceptable daily intake (ADI) of EDTA by a factor 11.4 or even higher.
- No negative health effects have been reported on the pharmaceutical use of iron EDTA in infants at levels exceeding the maximum ADI of EDTA by a factor 12 to 37.
- A daily intake of 5 mg Fe as iron EDTA, which is twice as high as the current maximum ADI of EDTA, would provide sufficient iron for children 6 to 24 months of age.
- A higher maximum ADI of EDTA could also improve zinc absorption.

## EDTA

EDTA is a synthetic chemical used in a wide range of applications (Anderson & Gaunt 1960). When dissolved in water, for instance in the form of disodium EDTA (Na_2_H_2_EDTA), EDTA molecules can form highly stable complex ions with two‐ and three‐valent metal ions. An EDTA molecule is comparable with a hand holding a tennis ball, where the tennis ball is the metal ion. All metal EDTA complex ions are negatively charged and highly soluble in water.

Many applications of EDTA were introduced to the market decades ago (Anderson & Gaunt 1960; Furia 1964). For instance, solutions containing EDTA are highly effective in dissolving deposits of calcium salts and this unique property has led to a widespread use in cleaning formulations. EDTA is used in the manufacture of paper to inactivate traces of manganese, which can catalyse the rapid decomposition of the wood pulp bleaching agent, hydrogen peroxide. Additionally, metal EDTA salts of iron, manganese, zinc and copper are used in growing crops as a highly effective source for these trace metals. Various EDTA derivatives are used in cosmetics (Lanigan & Yamarik 2002), and calcium EDTA and disodium EDTA have been approved as additives for a large number of food products to prevent rancidity and discolouration (Furia 1964).

### Multi‐year animal studies

From the large number of publications on EDTA, only three multi‐year studies in rats were found (Table 1). One of these studies was a PhD thesis (Yang 1952), of which only a brief summary is available in the public domain (BIBRA 1964), with no indication whether the data have been peer‐reviewed. However, these data are referred to in regulatory documents such as a recent Generally Recognized As Safe (GRAS) Notice of the US Food and Drug Administration (FDA 2011). Yang maintained intake levels of EDTA in the feed at 0.5%, 1.0% and 5% disodium EDTA (Na_2_H_2_EDTA). Based on an assumed ratio of 20:1 of dietary level vs. daily intake per kilogram bodyweight (Oser *et al*. 1963), these disodium EDTA concentrations in the feed correspond to oral intake levels of 250, 500 and 2500 mg day^−1^ kgbw^−1^, respectively. An intake of 2500 mg day^−1^ kgbw^−1^ of disodium EDTA is equivalent to an intake of 7440 *μ*mol day^−1^ kgbw^−1^ of EDTA molecules. The experimental animals given the highest level of disodium EDTA suffered from continuous diarrhoea, consumed less feed than the lower dose groups and failed to produce litters. No other adverse health effects were detected in this experiment, including in the highest dose group.

**Table 1:** Overview of multi‐year studies in rats with high levels of oral intake of EDTA

Reference	EDTA		Highest dosage level		
	Chemical form	Molecular mass (in mg mmol^−1^)	In the feed	As mg day^−1^ kgbw^−1^ *	As *μ*mol day^−1^ kgbw^−1^
BIBRA 1964, ^†^	Na_2_H_2_EDTA	336	5%	2500	7440
			1%	500	1488
Oser *et al*. 1963	CaNa_2_EDTA	374	5000 ppm	250	668
NCI 1977	EDTA‐Na_3_H·3H_2_O	412	7500 ppm	375	912

*A conversion factor of 20 was used for concentration in the feed and the intake per day per kilogram bodyweight (Oser *et al*. 1963). ^†^For the 1952 Yang study (BIBRA 1964), the second highest dosage level has also been included.

In an extensive study (Oser *et al*. 1963), the highest intake level was 250 mg day^−1^ kg^−1^ of calcium EDTA (CaNa_2_EDTA), which is equivalent to 668 *μ*mol day^−1^ kgbw^−1^ of EDTA molecules. At this highest intake level, no adverse health effects were observed. This study was referred to by JECFA in 1973 in determining the maximum ADI of EDTA (JECFA 1974). Higher levels were not tested as these were known to cause diarrhoea in the experimental animals (Foreman *et al*. 1953). This detailed study was intended to prove the safety of calcium EDTA and disodium EDTA as food additives. By applying a safety margin of 100, JECFA arrived at the current value for the maximum ADI of EDTA of 2.5 mg day^−1^ kgbw^−1^, expressed as CaNa_2_EDTA (JECFA 1974). This is equivalent to 6.7 *μ*mol day^−1^ kgbw^−1^ of EDTA molecules.

An also extensive study by the US National Cancer Institute in 1977 used trisodium EDTA trihydrate (EDTA‐Na_3_H·3H_2_O) at 7500 ppm in the highest dose level (NCI 1977). This corresponds to 375 mg day^−1^ kgbw^−1^ and is equivalent to 910 *μ*mol day^−1^ kgbw^−1^ of EDTA molecules. This study focused on identifying potential carcinogenic properties of EDTA, but no carcinogenic or any other adverse health effects were detected.

### Other toxicological data from animal studies

The data above indicate that experimental animals tolerate an oral intake of around 600–700 *μ*mol day^−1^ kgbw^−1^ of EDTA molecules (∼250 mg day^−1^ kgbw^−1^ of calcium EDTA). At double this level (∼500 mg day^−1^ kgbw^−1^ of calcium EDTA), diarrhoea has been observed occasionally in experimental animals, but no other harmful health effects have ever been reported (Foreman *et al*. 1953).

At a level of 3% (30 000 ppm) disodium EDTA in the feed of gestational rats, severe malformation of offspring was observed, but at a level of 2% disodium EDTA, reproduction was impaired only slightly (Swenerton & Hurley 1971). This 3% addition level is equivalent to an oral intake of 1500 mg day^−1^ kgbw^−1^ or approximately 4500 *μ*mol day^−1^ kgbw^−1^ of EDTA molecules. For a pregnant woman weighing 60 kg, this would mean a daily intake of 90 g of disodium EDTA. These teratogenic (embryo‐damaging) effects in the gestational rats were fully reversed with the addition of 1000 ppm zinc (∼15 mmol kg^−1^) to the feed containing 3% disodium EDTA (∼90 mmol kg^−1^). At these extremely high intake levels, EDTA may very well have removed zinc from the body of these rats and severe zinc deficiency is a teratogenic condition. The addition of a relatively small amount of zinc to the feed in a molar ratio of EDTA to zinc of 6:1 prevented developmental damage to the rat offspring.

### EDTA in humans

Both calcium EDTA and disodium EDTA can be safely administered intravenously in humans. When present in the blood circulation, EDTA molecules can bind lead ions effectively (Rubin *et al*. 1953). Intravenous application of EDTA has been demonstrated to improve renal function and to slow down the progression of renal insufficiency in patients with a ‘high‐normal body lead burden’ (Lin *et al*. 2003).

When administered into the blood circulation, all EDTA molecules are bound to metal ions, such as calcium, iron and zinc. The resulting metal EDTA complex ions are not metabolised by the liver, and do not mimic any hormone in the human body (no activity as an endocrine disruptor). All metal EDTA complex ions are soluble in water and are cleared rapidly by the kidneys.

However, when disodium EDTA is injected at very high levels so that all metal ions in the blood circulation are bound by an EDTA molecule (above approximately 2500 *μ*mol L^−1^), the remaining EDTA molecules that are not bound to a metal ion can be expected to extract metal ions from nearby endothelial cells. Therefore, when applied intravenously at a level exceeding around 2500 *μ*mol L^−1^ in the blood circulation, disodium EDTA becomes toxic (FAO 1965a,b). This can occur, for example, when an adult dose of disodium EDTA for chelation therapy is given to a small child.

Unlike intravenous administration, this acute toxic affect is not displayed when, after oral intake, EDTA molecules enter the blood circulation via the gastrointestinal tract and are able to combine with a metal ion. On an assumption that a small child ingests 5.6 mg iron (100 *μ*mol) as iron EDTA and 5% of the EDTA molecules are absorbed from the gastrointestinal tract (Foreman & Trujilio 1954; Heimbach *et al*. 2000), 5 *μ*mol of EDTA molecules will enter the blood circulation. Assuming a total blood volume of 0.5 L in a small child, the concentration of EDTA molecules is at most 10 *μ*mol L^−1^. This is far below the danger threshold of 2500 *μ*mol L^−1^ stated earlier.

In the 1970s, there was concern in the United States that the presence of calcium EDTA and disodium EDTA in food might depress iron absorption as a consequence of strong binding of the intrinsic iron ions (Cook & Monsen 1976). Meantime, iron EDTA has emerged as an effective source of this mineral in food (Hurrell *et al*. 2010). Since the 1980s, the safety of the widespread use of these two food additives has not been questioned in relation to the risk of mineral deficiencies. In 1995, the EU approved calcium EDTA for a number of food products (European Community 1995). Whether extremely high levels of calcium EDTA and disodium EDTA in food could pose a risk of inducing mineral deficiencies in humans is not known, although with current guidelines they are more likely to enhance absorption of iron and maybe even of zinc (Davidsson *et al*. 1994; Hettiarachchi *et al*. 2004).

### Iron EDTA in food

When iron EDTA is consumed in food, the ferric ions remain bound to EDTA as ferric EDTA complex ions in the stomach and the small intestine, including the duodenum. EDTA molecules can bind to both ferric and ferrous ions. When bound to EDTA, the ferric form is strongly preferred from a thermodynamic point of view, but nevertheless reduction to the ferrous form is possible (Seibig & Van Eldik 1997). Unlike ferric EDTA complex ions, the stability of ferrous EDTA complex ions is relatively low under acidic conditions. The cell walls of duodenal cells contain the proteins DCytB (duodenal cytochrome B), which is a strong reducing agent, and DMT1 (divalent metal‐ion transporter 1), which allows specifically ferrous ions to permeate the cell wall into the cytoplasm. It is probable that the ferric EDTA complex ions first undergo reduction by DCytB to the corresponding ferrous EDTA complex ions and, subsequently, the DMT1 proteins detach the ferrous ions from their accompanying EDTA molecules and transport them to the cytoplasm. This process is driven by the low pH of the outer membrane microenvironment during this transport of ferrous ions (Gunshin *et al*. 1997). Animal experiments support this mechanism of a separation of the iron ions from the EDTA molecules prior to entering the blood circulation (Zhu *et al*. 2006).

An EDTA molecule that is left behind after having released an iron ion to an intestinal cell can pick up another iron ion in the lumen of the gastrointestinal tract and also present it to an intestinal cell. That iron ion could very well be one that had been precipitated by phytate. This mechanism is known as the shuttle effect of EDTA and is believed to enhance the effectiveness of iron absorption even further (Lynch *et al*. 1993).

The use of iron EDTA in food fortification has grown in the past 10 years (Bothwell & MacPhail 2004, Wreesmann 2009). Iron EDTA is used to fortify powdered beverages in Brazil and the Philippines (Mondelez International 2011), soy sauce in China (Chen *et al*. 2005), wheat flour in China (Sun *et al*. 2007) and in Kyrgyzstan (UNICEF 2009), maize and wheat flour in Tanzania and Kenya (Andang'o *et al*. 2007), and fish sauce in Cambodia (Kingdom of Cambodia 2012). The World Food Programme (WFP) has recently specified that iron EDTA is one of the iron compounds that should be added to special nutritious products, and depending on the type of food, another iron fortificant could be added to meet the required iron content without exceeding the maximum ADI of EDTA (WFP 2011). The US Agency for International Development (USAID) has included iron EDTA in various food specifications (USAID 2013). Iron EDTA is added to some micronutrient powders (Troesch *et al*. 2011; Macharia‐Mutie *et al*. 2012) and is also recommended for supplementary foods for the management of moderate acute malnutrition in infants and children 6–59 months of age (WHO 2012b).

### Pharmaceutical use of iron EDTA

**Figure 2:**
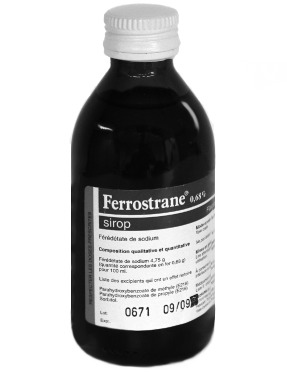
Ferrostrane, medicinal syrup based on sodium feredetate (iron EDTA) and sold in 250‐mL bottles in France.

In France, iron EDTA is approved as the active pharmaceutical ingredient for a drug with the trade name Ferrostrane (Fig. 2) as a medicinal syrup for infants and young children (Hodgkinson 1961; Kahn & Larsen 1980). This syrup contains 0.68% iron, which corresponds to ∼50 mg mL^−1^ of iron EDTA. This medicine has been in the market in France since the 1960s (HAS 2013) and is also commercially available in a number of other countries, including the UK (trade name Sytron), Sweden, Senegal, Algeria, Egypt and Pakistan (Akzo Nobel Functional Chemicals B.V., personal communication).

According to the package insert of Ferrostrane, the recommended intake is 5–10 mL day^−1^ for infants aged 1–6 months with a bodyweight of 5–8 kg and at the same dose for pregnant women, and 10–15 mL day^−1^ for children between 6 and 30 months (8–12 kg). In all cases, treatment duration is 3–6 months (ANSM 2012). An overview of the dosage recommendations for infants and young children, and the resulting intake of EDTA as iron EDTA is presented in Table 2.

**Table 2:** Recommended dosage on the package insert of Ferrostrane for young children

Age group in months	Bodyweight in kg	Recommended intake in mL day^−1^	Intake as iron EDTA		
			in mg day^−1^ *	in mmol day^−1^ ^†^	in *μ*mol day^−1^ kgbw^−1^
1–6	5–8	5–10	250–500	0.6–1.2	75–240
6–30	8–12	10–15	500–750	1.2–1.8	100–225

*Based on a content of 50 mg mL^−1^ of iron EDTA in the syrup (ANSM 2012). ^†^The molecular mass of iron EDTA (EDTA‐FeNa·3H_2_O) is 421 mg mmol^−1^.

A dosage of 10 mL day^−1^ contains 68 mg of iron, which is equivalent to 1.2 mmol (1200 *μ*mol). For an infant with a bodyweight of 5 kg, this daily dose means an intake of 240 *μ*mol day^−1^ kgbw^−1^ of EDTA molecules. A dosage of 5 mL day^−1^ for an 8‐kg infant is equivalent to an intake of 75 *μ*mol day^−1^ kgbw^−1^.

The intake of 75–240 *μ*mol day^−1^ kgbw^−1^ of EDTA molecules is 12–37 times higher than the current maximum ADI of 6.5 *μ*mol day^−1^ kgbw^−1^ of EDTA molecules (JECFA 2007).

The chelates plant of AkzoNobel in Herkenbosch, the Netherlands, has been the sole supplier of the active pharmaceutical ingredient for Ferrostrane (sodium feredetate; iron EDTA) for more than 20 years. During this period, the company has never been informed of any adverse health effects (Akzo Nobel Functional Chemicals B.V., personal communication). This absence of reported adverse health effects, while not strictly a proof as safety, adds weight to the scientific evidence reported previously.

### Overview of EDTA intake levels

An overview of maximum intakes of EDTA expressed as EDTA molecules in *μ*mol day^−1^ kgbw^−1^ and as H_4_EDTA in mg day^−1^ kgbw^−1^ is given in Table 3 for observations in animal studies and in Table 4 for recommendations in humans. The highest dose level of the 1952 Yang study (7440 *μ*mol day^−1^ kgbw^−1^ of EDTA molecules) and a safety margin of 100 would result in a value of 74.4 *μ*mol day^−1^ kgbw^−1^ of EDTA molecules or 21.7 mg day^−1^ kgbw^−1^ as H_4_EDTA for maximum ADI of EDTA. This value is 11.4 times higher than the current maximum ADI of EDTA of 6.5 *μ*mol day^−1^ kgbw^−1^ of EDTA molecules (JECFA 2007). Based on the second highest dose level of the 1952 Yang study, the maximum ADI of EDTA would be 2.3 times higher than this limit, and based on the highest dose level of the 1977 NCI study 1.4 times higher.

**Table 3:** Maximum intake levels of EDTA administered in animal studies

Reference	Expressed as EDTA molecules in *μ*mol day^−1^ kgbw^−1^	Expressed as H_4_EDTA in mg day^−1^ kgbw^−1^	Normalised to current maximum ADI of EDTA (JECFA 2007)
BIBRA 1964	7440	2170	1140
	1488	434	228
NCI 1977	912	265	140
Oser *et al*. 1963	668	195	103

ADI, acceptable daily intake. For the 1952 Yang study (BIBRA 1964), the second highest dosage level has also been included.

**Table 4:** Maximum intake levels of EDTA recommended for human consumption

Reference	Expressed as EDTA molecules in *μ*mol day^−1^ kgbw^−1^	Expressed as H_4_EDTA in mg day^−1^ kgbw^−1^	Normalised to current maximum ADI of EDTA (JECFA 2007)
JECFA 2007	6.5	1.9	1.0
ANSM 2012	240.0	70.0	37.0
Wreesmann 2007	17.9	5.2	2.8
This publication	74.4	21.7	11.4

ADI, acceptable daily intake.

In response to the announcement of a safety re‐evaluation of iron EDTA by JECFA in 2007, AkzoNobel submitted a dossier with the proposal to raise the maximum permitted level of iron as iron EDTA for 6‐ to 24‐month‐old infants and young children to 5 mg day^−1^ (Wreesmann 2007). This amount is equivalent to 89 *μ*mol day^−1^ of iron and the same amount of *μ*mol day^−1^ of EDTA molecules. For a 5‐kg infant, this intake of 5 mg day^−1^ iron as iron EDTA is equivalent to 17.9 *μ*mol day^−1^ kgbw^−1^ of EDTA molecules, which is 2.8 times higher than the current maximum ADI of EDTA. The AkzoNobel dossier advocated that this maximum oral intake of 5 mg day^−1^ iron as iron EDTA should be considered independently of the presence of calcium EDTA and disodium EDTA in food. The intake of these two EDTA‐based food additives could remain compliant with the maximum ADI of EDTA as defined by JECFA (JECFA 1974).

The safety re‐evaluation process resulted in a monograph (Pronk & Schlatter 2008), in which JECFA outlined that they could not support a rise of the maximum ADI of EDTA. However, the previous JECFA statement on iron EDTA of 1999 (JECFA 2000) was revised from ‘could be considered safe’ to ‘is suitable’, which is clearer confirmation of its safety for human use. Furthermore, two ambiguous sentences were removed from the previous statement ‘when used in supervised food fortification programmes in response to a need for iron supplementation in a population as determined by public health officials’ and ‘such programmes would provide a daily iron intake of approximately 0.2 mg kg^−1^ of body weight’. This revised JECFA statement has greatly supported the acceptance of iron EDTA for nutritional purposes by decision makers and Health Authorities in the EU (EFSA 2010; EU 2010; EU 2011) and in India (Gazette of India 2011).

In their safety evaluation report, JECFA rounded the figure of 1.95 mg day^−1^ kgbw^−1^ for the maximum ADI of EDTA expressed as H_4_EDTA (corresponding with 2.5 mg day^−1^ kgbw^−1^ of CaNa_2_EDTA) downwards to 1.9 rather than upwards to 2.0 (JECFA 2007). As a result, the normalised value for the principal animal study in Table 3 is not equal to the theoretical value of 100, but to 103.

## Discussion

It is now generally accepted that the EDTA molecules in iron EDTA fulfil a unique, positive role in delivering the iron ions from phytate‐rich diets to the human body (Hurrell *et al*. 2010). Nevertheless, there is concern that EDTA molecules may additionally have some negative impact on health. Although not justified by observations in high‐dosage animal experiments, this concern may have been aggravated by the emphasis on the maximum ADI of EDTA in regulatory documents on iron EDTA (Whittaker *et al*. 1993; Heimbach *et al*. 2000). Another view is that EDTA may represent a rare example of a synthetic chemical that is harmless to human health (Rowbury 2011). Besides a beneficial effect on iron absorption, EDTA in food could also contribute to lowering the absorption of lead (Flanagan *et al*. 1982; Kim *et al*. 2009) and to enhancing the uptake of zinc (Davidsson *et al*. 1994; Hettiarachchi *et al*. 2004).

The three long‐term animal studies summarised in Table 1 revealed no significant adverse health effects, except at the highest level (7440 *μ*mol day^−1^ kgbw^−1^) where continuous diarrhoea, reduced appetite and no offspring were reported (BIBRA 1964). Nevertheless, extensive examination of the organs and tissues of the test animals did not reveal any change compared with the control group. The second highest dosage (1488 *μ*mol day^−1^ kgbw^−1^) showed no adverse effects. The adverse effects at the highest dosage level could be due to the very high intake of sodium instead of the high EDTA intake. The exposure to sodium ions from the addition of 7440 *μ*mol day^−1^ kgbw^−1^ of disodium EDTA was 14 880 *μ*mol day^−1^ kgbw^−1^. This corresponds to 870 mg day^−1^ kgbw^−1^ NaCl, which is equivalent to 52 g daily salt intake for a 60‐kg adult. Assuming that the adverse effects were due to the high intake of sodium instead of EDTA, the highest dose would be safe and could be used as a basis for the determination of the maximum ADI of EDTA.

Two of the three long‐term studies reported positive health effects apparently induced by EDTA intake. The NCI study (NCI 1977) concluded that ‘the rats exhibited a negative dose‐related trend in survival’, indicating the rats tended to survive longer on feed containing more EDTA. In the summary of the PhD thesis, Yang stated: ‘The highest mortality occurred in group I [0 = control] and, in decreasing order, in groups II [0.5% disodium EDTA] and III [1.0% disodium EDTA]. There were no deaths in group IV [5.0% disodium EDTA]’ (BIBRA 1964).

JECFA during their 17th meeting in 1973 (JECFA 1974) considered only the study of Oser *et al*. 1963. Had they considered other long‐term animal studies (see Table 1), they might have proposed a higher value for the maximum ADI of EDTA. If the data from the NCI study (NCI 1977) had been available when JECFA defined the maximum ADI of EDTA in 1973, it would now likely have been 9.1 *μ*mol day^−1^ kgbw^−1^. This is 1.4 times higher than the current level of 6.5 *μ*mol day^−1^ kgbw^−1^. While an increase in the maximum ADI of EDTA by a factor 1.4 would be beneficial, it would still probably not meet the daily needs for iron via iron EDTA for 6‐ to 24‐month‐old children. Had JECFA in 1973 used the second highest dosage group of Yang 1952 (BIBRA 1964), which showed no adverse health effects, the maximum ADI of EDTA would now be 14.9 *μ*mol day^−1^ kgbw^−1^, which is 2.3 times higher than the current maximum. This increase by a factor 2.3 would meet the iron requirements of 6‐ to 24‐month‐old children. Nevertheless, if JECFA in 1973 had used the data of the highest dosage group in the Yang study (BIBRA 1964), which resulted in continuous diarrhoea, reduced appetite and no offspring (but no organ changes), the maximum ADI of EDTA would have been 74.4 *μ*mol day^−1^ kgbw^−1^. This is equivalent to 21.7 mg day^−1^ kgbw^−1^ and 11.4 times higher than the current 1.9 mg day^−1^ kgbw^−1^ (JECFA 2007), and about one‐third of the highest intake level of EDTA molecules as provided by Ferrostrane.

Recently, US FDA affirmed a self‐declared determination as GRAS by Del Monte Foods for the use of disodium EDTA and calcium EDTA in packaged cooked sweet corn products (FDA 2011). In order not to exceed the current maximum ADI of EDTA, calcium EDTA and disodium EDTA were set at a maximum level of 200 and 165 ppm, respectively. To support the safety assessment, the dossier submitted also referred to the Yang animal study of 1952 (BIBRA 1964). As already stated, the ratio of the level in feed in mg kg^−1^ to intake level in mg day^−1^ kgbw^−1^, also known as conversion factor, is generally assumed to be 20:1 (Oser *et al*. 1963). However, the applicants took a conversion factor of 10 rather than 20. In referring to the Yang study of 1952 (BIBRA 1964), the dossier states: ‘Rats were fed a diet containing 0, 0.5, 1.0, or 5.0% disodium EDTA (equivalent to approximately 0, 500, 1000, or 5000 mg kg^−1^ day^−1^)’. An intake of 5000 mg day^−1^ kgbw^−1^ is twice as high as the value of 2500 mg day^−1^ kgbw^−1^ (Table 1) and thus the assumed intake is twice as high as that outlined earlier. With the same safety margin of 100, but with a conversion factor of only 10, the maximum ADI of EDTA becomes 149 *μ*mol day^−1^ kgbw^−1^. This is equivalent to 43.5 mg day^−1^ kgbw^−1^ of EDTA and 23 times higher than the current 1.9 mg day^−1^ kgbw^−1^ (JECFA 2007). Although the use of a conversion factor of 10 rather than 20 still needs a careful defence, intakes as high as 21.7–43.5 mg day^−1^ kgbw^−1^ of EDTA as iron EDTA are similar to those achieved through the nearly 50 years' use of Ferrostrane.

An intake of 5 mg iron as iron EDTA per day, either through fortification of complementary foods or in the form of a home fortificant, such as micronutrient powders or lipid‐based nutrient supplements, is considered necessary to ensure adequate iron absorption from the complementary feeding diet of young children (6–24 months of age), especially when largely based on plant source foods rich in phytate. While encouraging results have been reported with the addition of 2.5 mg iron as iron EDTA to the diet of young children (Troesch *et al*. 2011; Macharia‐Mutie *et al*. 2012), this was under controlled circumstances. In both studies, the iron EDTA was in the form of a micronutrient powder that provided other vitamins and minerals to reduce nutritional anaemia, and the micronutrient powder in the 2011 Troesch study also contained phytase, which degraded phytic acid (Troesch *et al*. 2009). In a study using a diet rich in both phytate and polyphenols, iron EDTA was shown to be effective, but the dose level was 10 mg iron as iron EDTA per meal, three times a week (Abizari *et al*. 2012).

In summary, based on the knowledge of how EDTA functions in the human body, the results of long‐term animal studies, and the nearly 50 years' use of Ferrostrane, a rise of the maximum ADI of EDTA can be considered safe. Based on the NCI study (NCI 1977), the maximum ADI of EDTA can be raised to 9.1 *μ*mol day^−1^ kgbw^−1^, which is 1.4 times higher than the current level. Based on the PhD thesis of Yang 1952 (BIBRA 1964), and the dosage level that did not show any adverse health effects, the maximum ADI of EDTA would be 14.9 *μ*mol day^−1^ kgbw^−1^, which is 2.3 times higher than the current maximum. Based on the highest dosage group of Yang's study (BIBRA 1964), which resulted in continuous diarrhoea, reduced appetite and no offspring (but no organ changes), the maximum ADI of EDTA would be increased to 74.4 *μ*mol day^−1^ kgbw^−1^, 11.4 times than the current maximum ADI of EDTA but only about one‐third of the highest exposure level to EDTA molecules as provided by Ferrostrane.

For a 5‐kg infant, this regulatory change to a maximum ADI of EDTA of 14.9 *μ*mol day^−1^ kgbw^−1^ would allow a daily intake up to 5 mg Fe as iron EDTA rather than the currently permitted level of maximum 2.2 mg. With respect to the fortification of complementary foods for infants and young children aged 6–24 months, an addition level ensuring a daily intake of 5 mg Fe as iron EDTA is likely to be sufficient to allow adequate iron absorption and would be entirely safe.

## Source of funding

Akzo Nobel Functional Chemicals B.V. provided funding for writing this article.

## Conflicts of interest

The author is employed by the chemical company AkzoNobel. The Business Unit Akzo Nobel Functional Chemicals B.V. is a major global manufacturer of food‐grade EDTA products, including iron EDTA and world's largest producer of iron EDTA for agricultural purposes.
